# Oxygen blenders for prevention of retinopathy of prematurity are highly cost-effective in Uganda

**DOI:** 10.3389/fmed.2026.1715127

**Published:** 2026-03-27

**Authors:** Sean R. Smith, Victoria Nakibuuka, K. Davina Frick, Yvonne E. Vaucher, Rhea Gandhi, Michael P. Blair, Anna L. Ells, Rebecca Lusobya, Lucy Namakula, Iddi Ndyabawe, Denis Erima, Sherwin J. Isenberg, Sarah H. Rodriguez

**Affiliations:** 1 Pritzker School of Medicine, University of Chicago, Chicago, IL, United States; 2 St. Francis Hospital Nsambya, Kampala, Uganda; 3 Department of Health Policy and Management, Johns Hopkins Bloomberg School of Public Health, Baltimore, MD, United States; 4 Johns Hopkins University Carey Business School, Baltimore, MD, United States; 5 Division of Neonatology, Department of Pediatrics, University of California, San Diego, San Diego, CA, United States; 6 UC Irvine School of Medicine, University of California, Irvine, Irvine, CA, United States; 7 Retina Consultants Ltd., Des Plaines, IL, United States; 8 Department of Ophthalmology and Visual Science, University of Chicago Medicine, Chicago, IL, United States; 9 Calgary Retina Consultants, Department of Surgery, University of Calgary, Calgary, AB, Canada; 10 Mulago National Referral Hospital, Kampala, Uganda; 11 Department of Ophthalmology, School of Medicine, College of Health Sciences, Makerere University, Kampala, Uganda; 12 Department of Ophthalmology, King Ceasor University, Kampala and Kisubi Hospital, Entebbe, Uganda; 13 Lubaga Hospital, Kampala, Uganda; 14 Masaka Regional Referral Hospital, Masaka, Uganda; 15 Jules Stein Eye Institute, University of California, Los Angeles, Los Angeles, CA, United States

**Keywords:** cost-effectiveness, cost-utility, health economics, oxygen blender, preterm, prevention, retinopathy of prematurity, Uganda

## Abstract

Blended oxygen has been shown to reduce severe retinopathy of prematurity (ROP) incidence worldwide, and more recently in Uganda. Although it has been established that ROP-driven visual impairment has a substantial societal burden and that screening and treatment programs are cost-effective in low- and middle-income countries, there is limited evidence for the economic impact of prevention with appropriate oxygen management equipment (OME) in these regions. We performed a cost-utility analysis of OME implementation comparing variations ranging between most and least favorable values. Disability-adjusted life years (DALYs) were calculated with a 3% discount rate. Calculations assumed a 62.1% incidence of visual impairment in untreated severe ROP and a disability weight of 0.184 for severe visual impairment due to ROP. One set of OME costs between $677.50–$1,844.11 USD in Uganda. We found that the cost to prevent one case of ROP-driven severe visual impairment in Uganda ranges from $25.55–$543.99, with a base case (best estimate) of $168.92 per case averted. This reflects 5.18 DALYs averted over the 58-year average Ugandan lifespan and a cost of $4.97–$104.98 to avert one DALY, with a base case of $32.60 per DALY averted. In comparison, prevention of malaria via mosquito net distribution was estimated in a 2020 meta-analysis to cost $50 for every DALY averted. The high-end estimate is roughly 10% of the annual gross domestic product per capita ($1,002.30). Therefore, OME is a cost-effective means to prevent severe visual impairment among infants in Uganda. Implementation of OME in Uganda would also reflect a clear ethical and humanitarian success.

## Introduction

Retinopathy of prematurity (ROP) is a leading cause of infant blindness and severe vision impairment worldwide (1). In the “first wave” of ROP, concentrated in the United States and Western Europe in the 1940s and 1950s, excessive supplemental oxygen was identified as a key risk factor (2). Following implementation of strict oxygen protocols, ROP was minimized until the “second wave” of ROP arose due to improved survival among exceptionally premature babies who were, secondary to the expanding threshold of viability, at higher risk of developing various diseases of extremely preterm birth. The “third wave” has been concentrated in low- and middle-income countries (LMICs) and affects premature infants of greater birth weights and gestational ages, suggesting that the third wave, like the first, may be due to lack of modern equipment to safely regulate oxygen (3, 4).

In 2008, it was estimated that at least 50,000 children worldwide, mostly in middle-income countries, were severely visually impaired or blind due to ROP (2). In 2010 alone, an estimated 20,000 infants became blind or severely visually impaired due to ROP, and 12,300 more developed mild/moderate visual impairment. At the time, Sub-Saharan Africa (SSA) did not have a high prevalence of ROP because neonatal care was not well developed and overall survival among preterm infants was low. However, with advances in neonatal care, this third wave has now expanded to SSA (4). In particular, hospitals in Uganda are demonstrating improved survival among preterm infants, which is contributing to an increased incidence of ROP, and therefore a greater need for prevention and treatment (5–7).

In addition to the individual and family cost of blindness, increasing rates of untreated ROP have significant economic consequences worldwide. In Mexico, for example, lifetime employment losses and caretaker losses associated with one blind individual were estimated to be $142,172 USD and $305,584 USD, respectively (8). Comprehensive programs to screen for and treat ROP, known to be cost-effective in the United States (9), were projected in a 2022 meta-analysis to be cost-effective in LMICs (10). In 2012, the lifetime savings associated with laser treatment for one case of ROP in Peru was estimated to be $195,297 (11). In SSA, ROP screening and anti-VEGF therapy in Rwanda was estimated to result in a potential lifetime cost savings of $5,180,108 for an entire one-year birth cohort (12).

However, these previous cost-effectiveness studies have largely focused on screening and treatment for ROP, rather than prevention. After prevention of preterm birth, the primary strategy for ROP prevention in preterm infants is the use of the appropriate concentration of oxygen to maintain a physiologically safe level of oxygen saturation (13). Oxygen blenders allow providers to control the concentration of oxygen being administered. Non-invasive pulse oximeters estimate the infant's oxygen saturation in real time, allowing hospital staff to titrate the oxygen concentration to an appropriate physiologic level. Together, these two pieces of oxygen management equipment (OME) reduce the incidence of conditions associated with hyperoxia in newborns such as ROP, bronchopulmonary dysplasia, and intraventricular hemorrhage (14). The use of this equipment is standard practice in neonatal intensive care units (NICUs) in the United States and Europe, but NICUs in LMICs often lack access to this equipment, especially those in SSA, where it was recently reported that the median number of neonatal units that could provide blended oxygen was zero (15). In this study, we explore the cost-effectiveness of OME for the prevention of ROP in Uganda as one example of a strategy that is applicable to SSA broadly, aiming to demonstrate that prevention of ROP-associated visual impairment will prove cost-effective for LMICs in the long term.

## Materials and methods

### Definition of terminology

*Status quo*: the current state of most preterm care in SSA without access to blended oxygen (7).

*Base case*: the best estimate of the cost-utility of OME implementation using point estimates for cost, efficacy, etc.

*Most favorable*: the estimate of cost-utility which assumes every variable is at its maximally advantageous value (described below).

*Least favorable*: the estimate of cost-utility which assumes every variable is at its maximally disadvantageous value (described below).

*Oxygen-associated ROP (OA-ROP)*: retinopathy of prematurity related to excessive oxygen administration in LMICs (3).

### Sensitivity analysis

We aimed to calculate varying figures for cost-effectiveness using the most favorable, least favorable, and best estimates of figures available in the literature. Most favorable values included, for example, low costs for OME and a high turnover for infants being treated with blended oxygen. Least favorable values included high costs for OME and long usage time for a single infant on OME (Tables 1, 2). The base case represents the closest estimates to actual costs in Uganda.

**Table 1:** Estimates (yearly totals) used for the cost-utility analysis.

**Assumption**	**Blenders**	**Estimate (*N*)**	**Estimate (%)**	**Reference**
Preterm birth rate	–	171,275/1,712,75	10%	WHO (17)
Preterm at-risk for ROP (% < 34 weeks) (N)	–	51,383/171,275	30%	Stewart (19)
At-risk survivors (N)	–	35,865/51,383	69.8%	Kirabira (20)
At-risk survivors w/sROP w/no blenders (N)	–	2,511/35,865	7%	Nakibuuka (24), Vinekar (23)
At-risk survivors w/sROP w/70% OME coverage (N)	–	879/25,106 + 753/10,759 (1,632/35,865)	3.5%, 7%	Derived value
Cases sROP w/SVI w/no screening or treatment (N)	w/no OME	1,559/2,511	62.1%	CRYO-ROP (25)
	w/70% OME	1,013/1,632	62.1%	
Surviving at-risk screened (N)	-	19,367/35,865	54%	Nakibuuka (24)
Screened w/sROP w/no blenders (N)	–	1,356/19,367	7%	Nakibuuka (24), Vinekar (23)
Screened w/sROP w/70% OME coverage (N)	–	474/13,557 + 407/5,810 (881/19,367)	3.5%, 7%	Derived value
Cases screened sROP w/SVI w/screening + treatment (N)	w/no OME	14/1,356	10% reactivation, 20% lost to follow-up, 50% progression to SVI^†^	Best-estimate
	w/70% OME	9/881		
Days on OME/infant	–	5 days	3–7 days	Ndyabawe (18)
Equipment cost (USD)	–	$1,341.25	$677.50–$1,844.11	Neotech (64), Lifebox (65), Vayu (29)
Equipment lifespan	–	5 years	3–10 years	Best estimate

The number of cases of screened severe ROP (sROP) represents “known” cases after implementation of a screening program. It does not account for unscreened or “unknown” cases.

^†^best estimates for these figures. There is a lack of sufficient data to calculate the rate of SVI with reactivation that goes untreated.

**Table 2:** Cost-utility analysis.

**Variable**	**Base case (medical air)**	**Base case (no medical air)**	**Most favorable case**	**Least favorable case**
OME cost	$1,341.25	$677.50	$677.50	$1,844.11
OME lifespan (years)	5	5	10	3
Length of treatment with OME (days)	5	5	3	7
Infants/year	73	73	122	52
Infants/OME lifespan	365	365	1220	156
Risk of SVI	2.2%	2.2%	2.2%	2.2%
Cost to prevent one case of SVI	$168.92	$85.33	$25.55	$543.99
DALYs averted/life	5.18	5.18	5.18	5.18
Cost/DALYs averted	$32.60	$16.52	$4.97	$104.98

### Clinical parameters

The number of total live births in Uganda in 2023 was estimated at 1,712,750 (16). The rate of preterm birth in Uganda in 2022 was 10% (Table 1) (17). There is no national data or estimate available for the proportion of preterm infants that are less than 34 weeks' gestational age in Uganda (and therefore at higher risk of developing ROP). The first major ROP study from Uganda, which included the two major national referral hospitals, reported that 62% of preterm infants admitted to neonatal units were less than 32 weeks' gestational age (18). This likely over-represents the proportion of preterm infants in the country that are less than 32 weeks' gestational age as these infants are more likely to be admitted to a neonatal unit. Given this, and the fact that we aimed to capture infants less than 34 weeks' gestational age, we chose to use a proportion of 30% from an American study (19). As this gestational age group in Uganda may make up a smaller proportion of all preterm infants as survival among this group is higher in the United States, we also assume a 30.2% mortality rate among these infants based on a subset of preterm infants in a prior Ugandan study on infant mortality from 2020 (20).

We therefore estimate that there are 171,275 preterm births per year in Uganda of whom 30% (*N* = 51,383/yr) are less than 34 weeks' gestation. Including the 30.2% mortality rate (20), 69.8% (*N* = 35,865/yr) of the infants less than 34 weeks' gestation are at higher risk for developing ROP if oxygen cannot be blended (21). We estimate that without blended oxygen, the rate of severe ROP [Type 1 ROP or worse as defined by the ET-ROP study (22)] among at-risk premature infants in neonatal units in Uganda is 7%, based on the KIDROP study (23). When blended oxygen is used, we estimate that the rate of severe ROP falls to 3.5%, based on our prior study which observed a roughly 50% decrease in the rate of severe ROP after OME implementation (24). We explain in the Discussion that data from the KIDROP study, rather than local data, was used for the baseline given the extensive experience and patient volume in the KIDROP study from another country with extensive experience screening and treating patients in the 3^rd^ epidemic of ROP.

We assume that 62.1% of all the cases of severe ROP will be severely visually impaired or blind with no treatment, based on 10-year visual outcomes following patients from the CRYO-ROP (CRYOtherapy for Retinopathy Of Prematurity) study (25). This is higher than the original rate of adverse structural outcomes from the CRYO-ROP study because after 10 years, some patients without adverse structural outcomes still had unfavorable functional results and decreased visual acuity. Using 62.1% means that among at-risk preterm infants who receive 100% oxygen, 7% are assumed to meet criteria for treatment of ROP, and without treatment, approximately 4.3% (62.1% of 7%) may become severely visually impaired or blind. Similarly, among the infants managed with OME, 3.5% will need treatment for ROP, and without treatment, approximately 2.2% will be severely visually impaired or blind. In calculating the effect of a national screening and treatment program, we use the 54% screening rate observed at an experienced, high-acuity neonatal unit hospital in Kampala (24). The actual rate is currently much lower at the large public hospitals in Uganda. Additionally, we assume that most primary treatment will be intravitreal bevacizumab injection with a success rate of approximately 90%, based on the success rates of 85% and 96% seen in the ROPIC (Retinopathy Of Prematurity Injection Consortium) and BEAT-ROP (Bevacizumab Eliminates the Angiogenic Threat of Retinopathy Of Prematurity) studies, respectively (26, 27). We present our rationale for referencing these particular studies in the Discussion section.

### Cost parameters

As described in our previous study detailing the multidisciplinary intervention in Kampala, Uganda, donations were used for oxygen blenders, pulse oximeters, sensors and ancillaries (e.g., hoses, mounting equipment) (24). This intervention employed low-cost, high-impact equipment designed for use in low-income countries which offers significant cost savings compared to similar oxygen-blending and oximetry devices used in high-income countries while maintaining quality and reliability. The cost of that oxygen blender and associated equipment (Neotech, India) is $673 USD, which will serve as our base case in this analysis. This estimate represents a final cost that has been lowered with support from donations. In future transactions, lack of donations and payment of fees may increase the cost—Table 3 highlights ultimate costs for shipping the same equipment to other countries in SSA during similar initiatives in the past. In addition to the costs listed, for this analysis we add a 25% import duty to all equipment costs, per publicly available import duty information, to reflect total cost as paid in Uganda and comparable LMICs (28). We also note that in this analysis, we use a more recent estimate of $400 for oximeters and related equipment. Notably, Neotech blenders require medical air, which is not available in many Ugandan hospitals.

**Table 3:** Cost (USD) comparison for OME cost per unit.

**Country**	**Blenders**		**Oximeters**		**Full set of OME**	
	**Base Price**	**Final Cost**	**Base Price**	**Final Cost**	**Base Price**	**Final Cost**
Nigeria	$673	$807	$250	$280	$923	$1,087
Uganda	$673	$673	$250	$250	$923	$923
Rwanda	$673	$819	$250	$275	$923	$1,094

Variation in cost is secondary to import taxes and other fees.

Alternatively, since that study was published, a new low-cost oxygen blender which does not require medical air has become available (Vayu Global Health Innovations, USA). We obtained a cost estimate from a company in Uganda, which distributes devices such as this one, of $142. We clarify that this this price covers only the newly developed blender attachment to be used with a Vayu nasal cannula, exclusive of the full CPAP system (29, 30). This serves as our most favorable cost estimate. Because most neonatal units and lower-acuity health facilities in Uganda do not have medical air, we will also include a base case analysis that uses this price. (This base case will represent the best estimate for centers with or without medical air that use this device). In short, we include a most favorable scenario that includes this price and all other variables at their maximally advantageous values, as well as a second base case scenario (in addition to the base case described in the preceding paragraph) that uses this price but sets all other variables at the most realistic point estimates. While there are no current data that validate similar efficacy for the prevention of ROP with this system, for the purpose of this paper we assume equal efficacy to the traditional blender models.

We additionally include a least favorable cost estimate. We had previously contacted several American oxygen blender manufacturers (Maxtec, Precision Medical, Tri-State Biomedical, Tenacore, Tri-anim, FOBI Medical, Ohio Medical) for quotes when acquiring equipment for the hospital in Kampala; prices ranged from $599 to $1,785, with an average price of $1,075.29. These devices would be less likely to be deployed on a large scale in a low-resource setting, but we include this average price in our analysis to provide a range of feasible costs.

Low-cost pulse oximeters and related equipment cost $400 (Lifebox, U.K./Taiwan). Other consumables and ancillaries are included in this estimate. One oxygen blending device paired with one oximeter constitute one set of OME, with an OME cost range, including shipping and import fees, between $677.50 ($142 plus $400, with 25% tax) and $1,844.11 ($1,075.29 plus $400, with 25% tax) with a best estimate of $1,341.25 ($673 plus $400, with a 25% tax).

In a 2023 Ugandan study, the median number of days for preterm infants < 37 weeks on oxygen at two major referral centers was 5 days (ranging between 3 and 7 days) (18). Therefore, one set of OME could theoretically be utilized for an average of 73 infants per year (ranging between 52 and 122 infants per year). However, taking fluctuations in the census into account, assuming near-100% utilization of OME means that on approximately 99% of days, there will be at least one infant on unblended oxygen. Also, deployment of OME throughout Uganda would be unlikely to cover 100% of preterm infants born in a given year. As a result, we estimate a deployment rate of 70%, due to likely difficulty in deployment of OME to every neonatal unit, as well as non-coverage of non-neonatal units where some preterm infants are resuscitated.

Oxygen blenders typically last at least five years, and in this study we included a functional range between 3 and 10 years. As of publication of this work, the OME described in our previous study has been operational for five years (24). Routine maintenance and recalibration by hospital engineering staff should occur once per year, but represent minimal cost, which we have assumed to be negligible in this analysis. Per correspondence with the distributor in Uganda, the Vayu blender has a 3-year use guarantee but typically lasts longer than 5 years.

Lastly, we assume an average cost of $75 per infant for treatment with bevacizumab, ranging between $50–$100 in our experience. The cost per patient depends primarily on how many patients are able to be treated with bevacizumab from a single vial, which costs $345.06 in Uganda.

### Calculation of DALYs

We use the Global Burden of Disease Study 2021 disability weight (DW) of 0.184 for the 62.1% of infants with ROP that develop severe visual impairment (SVI), based on unfavorable structural outcomes among untreated patients with threshold disease in the CRYO-ROP study (25, 31, 32). When using this DW, we assume that in the base case scenario, no infants with ROP are treated, none will progress to complete blindness, and all infants who develop SVI will live to the average Ugandan life expectancy of 58 years (although it has been reported that over half of blind children in SSA will die within 1–2 years of becoming blind) (33).

Cost-utility analysis of OME implementation was performed comparing variations using most favorable values, least favorable values, and base case values. Disability-adjusted life years (DALYs) averted were calculated by applying a DW of 0.184 to a 58-year lifespan with 3% discounting (total DALYs averted are 5.18: see Equation 1 below). Calculations assumed 62.1% incidence of severe visual impairment in untreated ROP. We completed our cost-utility analysis in Microsoft Excel.


$$\begin{array}{l} DALYs\;=\;DW\;\left(0.184\right)\;\cdot \overset{57}{\underset{n=0}{\sum }}{\left(\frac{1}{1.03}\right)}^{n} \end{array}$$

Equation 1: Calculation of DALYs averted over 58-year lifespan with 3% discounting and DW of 0.184.

## Results

### Effects of prevention alone on the incidence of SVI due to severe ROP

**Figure 1:**
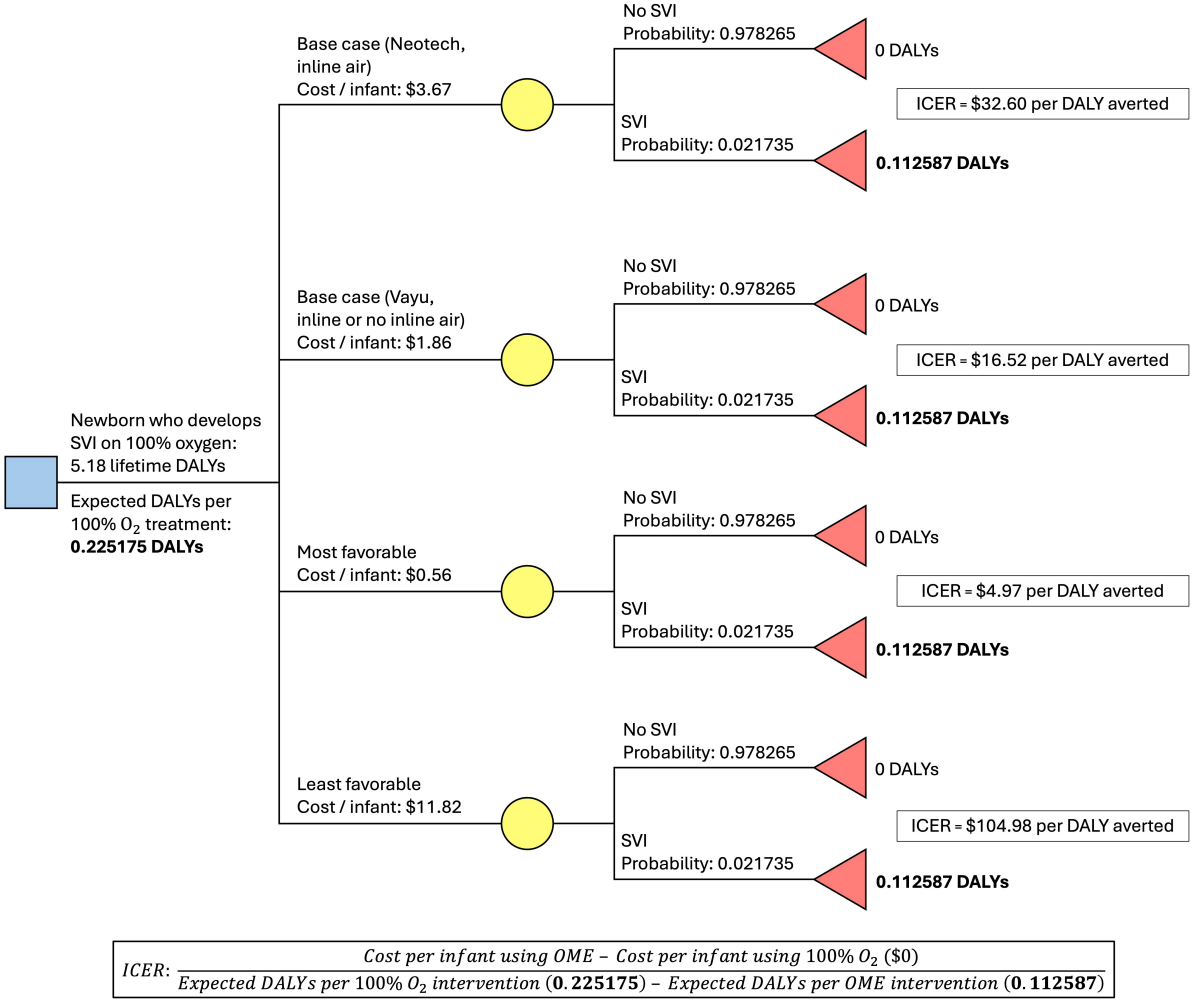
Decision tree for OME implementation. ICER (incremental cost-effectiveness ratio) represents the cost (in USD) per added benefit of a new intervention that replaces the existing intervention. Benefit in this analysis is measured in DALYs averted.

In the base case prevention-only scenario using the oxygen blender that requires medical air, the Neotech™ blender is used. The cost of one set of OME is $1,341.25 ($673 plus $400, with 25% tax). The equipment lasts for 5 years and is used to administer blended oxygen to 73 preterm infants per year, or 365 infants over the equipment's lifespan. The cost per infant using OME is therefore $3.67. In this scenario, approximately 2.2% (*N* = 7.93/365) of these infants will still develop SVI over 5 years compared to approximately 4.3% (*N* = 15.87/365) of preterm infants who do not receive blended oxygen (status quo). Therefore, per set of OME per 5 years, 7.94 infants who would have developed SVI do not. Based on the cost of $1,341.25 for one set of OME, the cost to prevent one case of SVI is $168.92. A disability weight of 0.184 for SVI due to ROP, applied over an average 58-year lifespan in Uganda with 3% discounting, equates to 5.18 DALYs averted over one lifespan, and the cost per DALY averted is therefore $32.60 (Figure 1).

In the base case prevention-only scenario using the oxygen blender that does not require medical air, the Vayu blender is used. The cost of one set of OME is $677.50 ($142 plus $400, with 25% tax). All other variables have the same value as the previous scenario, so the cost per infant using OME is therefore $1.86. A cost of $677.50 to prevent 7.94 cases of SVI equates to $85.33 to prevent one case of SVI. The same 5.18 DALYs are averted over the same 58-year lifespan, so the cost per DALY averted is therefore $16.52.

In the most favorable prevention-only scenario, the cost of OME is again $677.50, but lasts for 10 years, and 122 preterm infants per year receive blended oxygen from a single set of equipment (1220 infants over the equipment's lifespan). The cost per infant using OME is therefore $0.56; 26.52 infants who would have developed SVI do not, which equates to a cost of $25.55 to prevent one case of SVI. The same 5.18 DALYs are averted over the same 58-year lifespan, so the cost per DALY averted is therefore $4.97.

In the least favorable prevention-only scenario, the cost of OME is $1,844.11 ($1,075.29 plus $400, with 25% tax), lasts for only three years, and just 52 preterm infants per year receive blended oxygen from a single set of equipment (156 infants over the equipment's lifespan). The cost per infant using OME is therefore $11.82. 3.39 infants who would have developed SVI do not, which equates to a cost of approximately $543.99 to prevent one case of SVI. The same 5.18 DALYs are averted over the same 58-year lifespan, so the cost per DALY averted is therefore $104.98.

**Figure 2:**
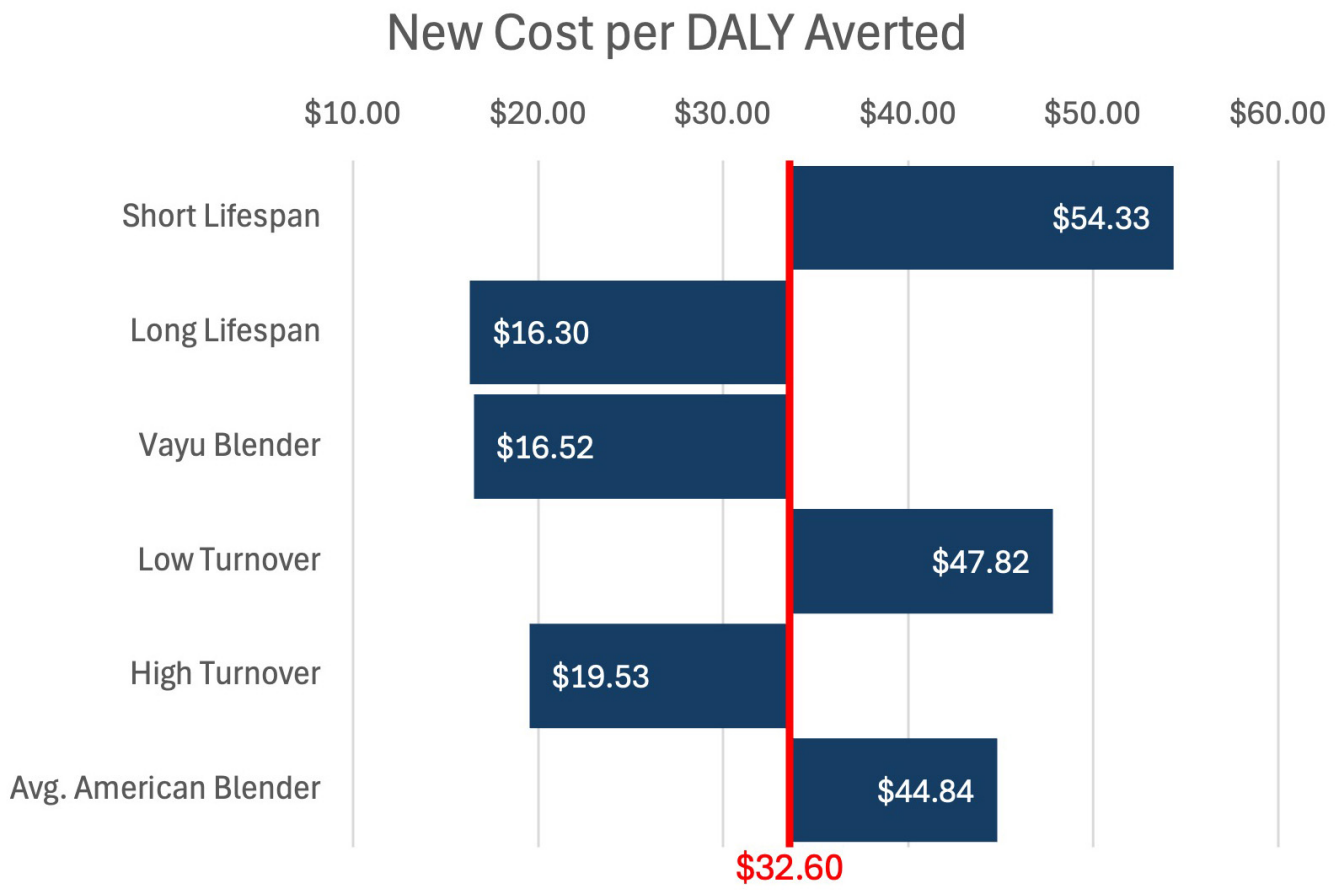
Univariate sensitivity analysis. $32.60 per DALY averted is the base case. Shortening the lifespan of the blender from 5 to 3 years resulted in the largest increase, in cost per DALY averted, from base case. Lengthening the lifespan of the blender from 5 to 10 years resulted in the largest decrease, in cost per DALY averted, from base case.

These results are presented in Figure 1 and Table 2. Figure 2 demonstrates a univariate sensitivity analysis of these variables (presented as change in cost per DALY averted given a change in one variable at a time).

### Effects of OME on prevention of ROP

If, among the entire annual Ugandan preterm birth cohort of 171,275, all high-risk infants less than 34 weeks' gestation who survive prematurity (*N* = 35,865) receive unblended, 100% oxygen, approximately 4.3% (*N* = 1,559) per year are likely to develop SVI due to OA-ROP in the absence of a comprehensive screening and treatment program. Conversely, with nationwide deployment of OME reaching 70% of these 35,865 infants (*N* = 25,106), a lower incidence of SVI due to severe ROP (approximately 2.2%) equates to 546 infants per year who could still develop SVI with improved oxygen management. In this scenario, 468 infants (4.3% of the 10,759 high-risk infants who receive 100% oxygen) would also still develop SVI (1,014 infants with SVI overall). Thus, deployment of OME could potentially prevent 545 cases of SVI annually. These numbers reflect reduction of risk of SVI by prevention alone, without a screening and treatment program in place.

### Combined effect of OME prevention and national screening and treatment

**Figure 3:**
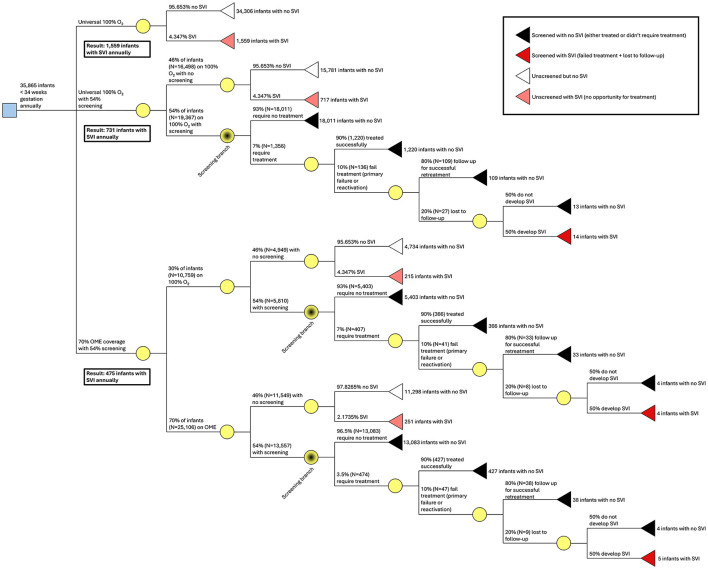
Decision tree for a national screening and treatment program. Three strategies are presented: 100% O_2_ for all 35,865 high-risk premature infants, vs. 100% O_2_ and 54% screening and treatment, vs. 70% OME coverage plus 54% screening and treatment. We note that these hypothetical scenarios are overestimates as not all high-risk premature infants in Uganda receive care in a neonatal intensive care unit.

We now explore the effect of screening and treatment for ROP in Uganda using the aforementioned estimates of a 90% success rate for bevacizumab injections and a 54% screening rate (24, 26, 27). We also assume that 80% of treated infants will complete long-term follow up (given that treated patients with established relationships are more likely to follow-up), and of the few infants who reactivate and are lost to follow up (2% of those treated, based on a 10% failure rate and a subsequent 20% lost to follow-up rate), 50% will develop SVI (given a lack of sufficient data to calculate the rate of SVI with untreated reactivation). If all 35,865 high-risk premature infants born at less than 34 weeks' gestation in Uganda annually and survive prematurity were to receive unblended 100% oxygen in a neonatal unit or lower-acuity health facility, 54%, or 19,367/35,865, could be screened (24). Of the 19,367 infants who are screened, 7% (*N* = 1,356) would be identified as needing treatment for ROP. With a success rate of 90% for bevacizumab treatment, 10% of those treated (*N* = 136) would still experience reactivation or treatment failure. (We note that not all high-risk premature infants in Uganda are born, or otherwise taken care of, in neonatal units and lower-acuity health facilities−35,865 is likely greater that the true number of surviving premature infants cared for in Ugandan healthcare facilities annually. We also note that far more ophthalmologists than are currently available in Uganda would be needed in order to screen this number of infants annually.) If we assume that 136 infants have severe reactivation, most would have returned and received additional treatment but 20% (*N* = 27) may be lost to follow-up, one half (*N* = 14) of whom would develop SVI (Figure 3). This screening and treatment program with no OME, which would have 1,356 initial treatments annually as well as 109 follow-up treatments annually, at $75 per treatment with a range of $50–$100, would have a total medication cost of $109,875 ($73,250–$146,500) per year.

If 70% of the 35,865 high-risk premature infants born at less than 34 weeks' gestation who survive prematurity were to receive blended oxygen, in the base case scenario, 19,367 infants would be screened (the same as in the last scenario). 70% of these screened infants (*N* = 13,557) would have received blended oxygen; 30% (*N* = 5,810) would have still received 100% oxygen. Of the 13,557 infants who received blended oxygen, 3.5% (*N* = 474) would need treatment for ROP. Likewise, 7% of the 5,810 screened infants who receive 100% oxygen (*N* = 407) would need treatment for ROP, totaling 881 infants who would need treatment. With a success rate of 90% for bevacizumab treatment, 10% of those treated (*N* = 88) would be at risk for vision loss considering the long term possibility of reactivation. Following the method above, 9 would still develop bilateral SVI. This screening and treatment program which includes 70% OME coverage, which would have 881 initial treatments annually as well as 71 follow-up treatments annually, at $75 per treatment with a range of $50–$100, would have a total medication cost of $71,400 ($47,600–$95,200) per year.

After implementation of OME, Implementation of OME within a nationwide screening and treatment program would result in 5 fewer cases of SVI every year among screened infants. Additionally, after implementation of OME, the number of treatments every year falls from the number of treatments needed every year to fall from 1,465 to 952 (513 fewer treatments) (Table 4). We note that we do not include here any cases of SVI prevented in unscreened infants; total numbers among both screened and unscreened infants are presented in Figure 3. (In the Discussion section, we explore the practicality of screening and treating high numbers of infants in Uganda.)

**Table 4:** Annual numbers of infants with SVI due to ROP in status quo and base case scenarios.

**Cohort**	No OME Status Quo^*^	**70% OME Base Case**
Surviving high-risk premature infants (*N*)	35,865	35,865
Infants screened (54%) requiring treatment (*N*)	1,356	881
Treated infants receiving retreatment (*N*)	109	71
Screened infants failing treatment and lost to follow-up w/SVI due to ROP (*N*)	14	9

^*^Status Quo reflects no equipment for blending oxygen and monitoring saturation, based on a study in which the median number of blenders among surveyed neonatal units in SSA was zero (15).

**Figure 4:**
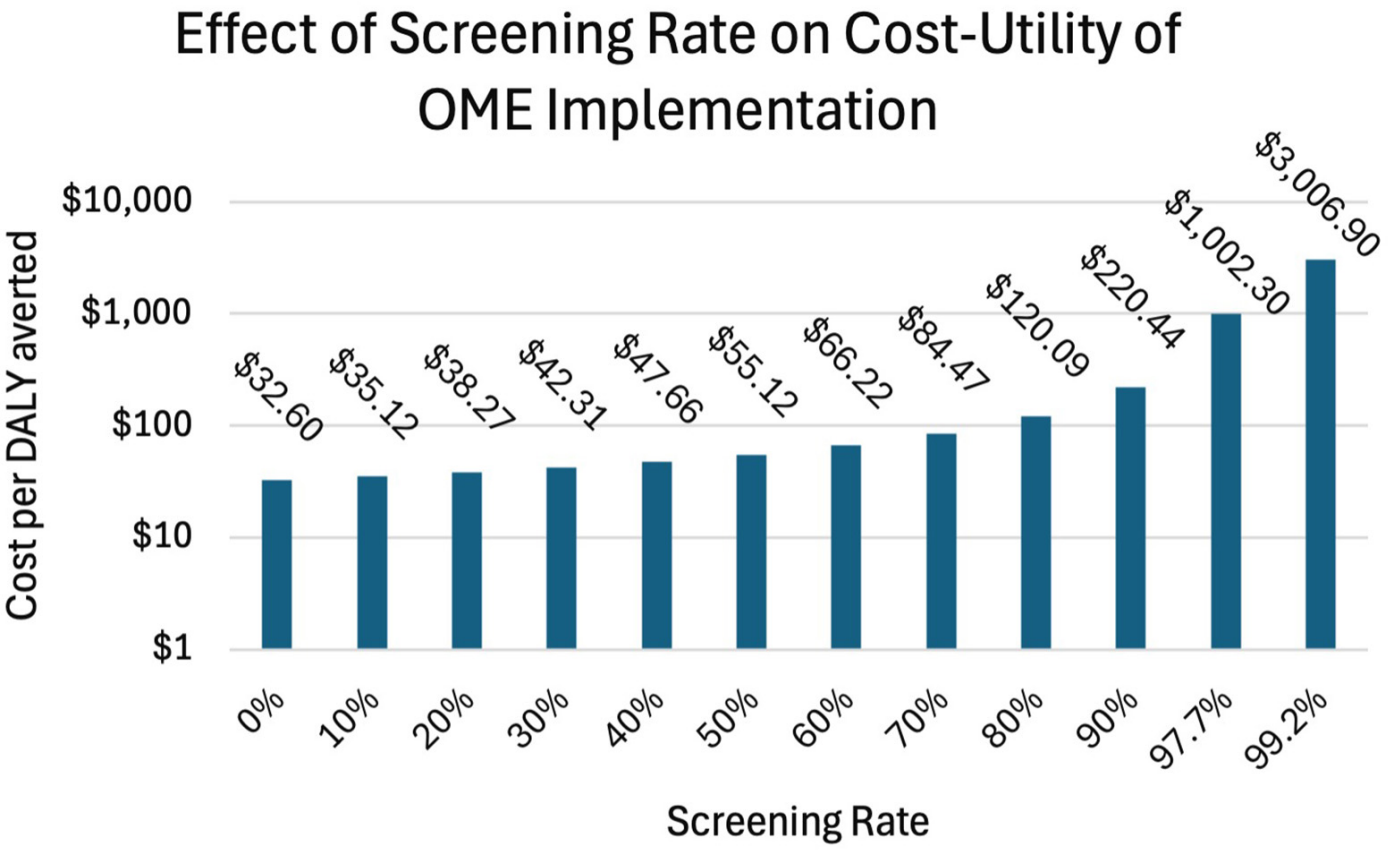
Effect of screening rate on cost-utility of OME implementation. A national screening and treatment program in place, with 100% oxygen being administered, is the status quo. At 0% screening, implementing OME results in an ICER of $32.60—this reflects the base case scenario in our analysis of implementing OME only. At 97.7% screening, a cost per $1,002.30 per DALY averted is reached, which was the average annual income in Uganda in 2023. At 99.2% screening, a cost of $3,006.90 per DALY averted is reached; three times the average annual income is a commonly used benchmark for the highest cost at which an intervention can still be considered cost-effective.

We also include a sensitivity analysis of the effect of variable screening rates on the overall cost-effectiveness of OME implementation (Figure 4). Here, administration of 100% oxygen with a national screening and treatment program functions as the status quo, and the addition of OME nationally is the base case. The screening is varied from 0 to 99.2% in increments of 10%, and the resulting ICER (cost per DALY averted as the result of adding OME) is shown. We note that we assume a linear relationship between screening rate and both DALYs averted by OME and treatment cost savings. The cost per DALY averted at screening rates of 0%, 50%, and 99.2% is $32.60, $55.12, and $3,006.90, respectively.

Of note, in Figure 4, the highest screening rate that we present is 99.2%. At this screening rate, the cost per DALY averted is $3006.90, which is three times the average annual income in Uganda, a commonly used WHO benchmark for the maximum cost per DALY at which an intervention can be considered cost-effective. At screening rates above this, implementation of OME would no longer be cost-effective. At a 100% screening rate, the cost per DALY becomes undefined, as implementing OME in such a scenario theoretically yields no additional health benefit—all infants who would have progressed to SVI due to 100% oxygen administration are successfully screened, treated, and followed perfectly to catch all cases of reactivation.

## Discussion

In this cost-utility analysis of prevention of ROP through OME implementation with oxygen blenders and oximeters, the cost to prevent one case of SVI ranges from $25.55–$543.99, with a base case of $168.92 if using the oxygen blender that requires medical air and $85.33 if using the oxygen blender that does not. Our previous single-center, cohort study in Kampala, Uganda found that the implementation of OME, along with an appropriate curriculum, care protocols, and ancillary equipment, significantly reduced the proportion of infants with severe ROP needing treatment from 14.9 to 6.9% (24). That study originally reported proportions of severe ROP 27.8% pre-intervention and 12.8% post-intervention; we multiplied these figures by the 54% screening rate in that study (as most infants who went unscreened were larger, healthier, and at lower risk of ROP) to reach an estimate of 14.9% and 6.9% incidence, respectively. Of note, the true rate of ROP among unscreened infants in that paper was likely not zero, but we assumed as such for the purpose of this analysis.

To more closely approximate the natural history of ROP, we used rates of severe ROP observed in the KIDROP study (23). The proportion of infants treated for ROP in our Uganda cohort, before and after (OME) implementation, were both higher than the corresponding rates reported in KIDROP (1.7%−7.1%), including government and private hospitals. This difference may be partially explained by context-specific clinical decision-making in Uganda, where treatment may be initiated because of anticipated loss to follow-up or inability to ensure timely surveillance. Although such decisions may be clinically appropriate in this setting, the risk of severe visual impairment from ROP in these cases may be less reliable. To avoid overestimating rates of childhood visual impairment and the associated cost-effectiveness of the intervention, we therefore used KIDROP-derived rates, leveraging the study's large sample size and the graders' extensive experience to better capture the natural history of ROP.

In Uganda, there are 21 neonatal intensive care units and many lower-acuity health facilities that are administering unblended oxygen to infants. Fewer than 10 of the 48 practicing ophthalmologists in Uganda are currently screening for and/or treating ROP. In addition, access to essential screening equipment, such as indirect ophthalmoscopes, specula, depressors, and eye drops, remains limited. Compounding shortages of ophthalmologists and equipment, intravitreal bevacizumab does not currently have an indication for ROP on the WHO Model List of Essential Medicines (34). Addition of bevacizumab for ROP to the List of Essential Medicines is needed to significantly drive down the cost paid by families who are currently responsible for buying bevacizumab, as hospitals in Uganda do not have it on formulary. A single vial of bevacizumab can potentially be shared, in our experience, among roughly five patients in hospitals without high volumes of ROP or diabetic retinopathy. Biosimilars are an emerging option that may enable more widespread access to treatment (35, 36) but need validation in this setting. Treatment does carry inherent risks (37–39) which are relatively low with bevacizumab, currently the most widely-used anti-VEGF agent in SSA. If other options such as aflibercept or ranibizumab were to be used, our overall calculations for the effect of a national screening and treatment program would change (40, 41). This is especially true in the case of ranibizumab, which has a relatively high reactivation rate.

We previously reported a 54% screening rate in a high-quality neonatal unit with an ROP program, indicating that interventions to prevent ROP in less-resourced hospitals are critically needed (24). Post-discharge screening rates at all sites are strongly influenced by geographic, socioeconomic, and cultural factors (e.g., distance, time required, availability of transport, cost, family priorities) which are difficult to address. However, efforts must be made to improve screening rates. Deploying digital imaging with telemedicine, as was accomplished in KIDROP and newer LMIC programs, should be prioritized alongside OME deployment (23). An intervention in Nigeria which included skills transfers and implementation of a national screening protocol proved effective in improving ROP screening coverage (42). (Six NICUs were providing ROP services in 2017, and this increased to 20 in 2018; the number of ophthalmologists confident in screening increased from 10 to 23 during this period.) Notably, lack of equipment to regulate oxygen was a challenge in all 20 NICUs. Other analyses have explored the exact cost-effectiveness of implementing national screening and treatment programs in SSA; a study in Rwanda estimated the cost of ROP screening and treatment with bevacizumab, both of which require ophthalmologists, at $738 per infant (12).

Implementation of OME will reduce the burden of SVI but will not eliminate it. Our calculated number of at-risk preterms who potentially require screening and treatment would overwhelm any national program. With improving survival, especially among earlier gestation preterm infants, the need for screening and treatment will continue to grow. In the long term, more ophthalmologists, effective screening programs, and ready access to anti-VEGF agents are essential. A broader preventative approach would likely prove to be more scalable and cost-effective across Uganda compared to a national screening and treatment program alone. Alongside prevention of ROP with OME, an important strategy to address the trend of increasing numbers of preterm infants is primary prevention of preterm birth. Alternatively, safely prolonging gestation or providing medical care that reduces the risk of associated problems will reduce the risk of ROP. These include various low-cost measures such as antenatal corticosteroids under specific circumstances, prevention of hypothermia, ensuring adequate nutrition, prevention of sepsis with hand hygiene, and antibiotic stewardship (43, 44). A 2017 study also showed that prevention of preeclampsia is associated with a reduced risk of ROP (45). Additionally, a 2012 study detailed a low-cost initiative to enroll premature infants into ROP screening programs immediately after birth upon weighing (46). These studies reflect a broad array of interventions that can be pursued before and after a premature infant is born in order to reduce the incidence of ROP.

Although we have focused our cost-utility analysis on Uganda as that is where our experience lies, this approach is applicable to SSA broadly and LMICs in general, and will be especially relevant in any country with a limited number of ophthalmologists relative to the annual number of premature infants treated with oxygen. We note that, as shown in Figure 4, a comprehensive screening and treatment program would decrease the utility of OME, as preventing ROP with OME would avert the costs associated with treatment rather than preventing SVI, assuming that screening and treatment substantially reduces the incidence of SVI. In settings with limited or absent screening, OME implementation results in a lower cost per additional DALY averted and prevents SVI. Conversely, in NICUs with near-universal screening and highly efficacious treatment, OME implementation would yield minimal additional health benefit; whether they received blended or 100% oxygen, all infants all infants who require treatment would receive it. As such, implementation of OME would not greatly reduce the total number of cases of SVI. Rather, in a scenario with high rates of screening, OME—by reducing the number of treatments needed and likely decreasing the duration of oxygen administration—functions primarily as a cost-saving measure.

However, the apparent cost-effectiveness of prevention is mathematically reduced only under the unrealistic assumption that all, or nearly all, infants are screened and treated—an ideal that is not achieved even in high-income settings such as the United States, where retinopathy of prematurity remains the leading cause of childhood blindness in recent data from the IRIS registry (47). In addition, unblended oxygen exposure is a well-established contributor to other conditions that cause neonatal mortality and lifelong morbidities such as bronchopulmonary dysplasia and neurodevelopmental impairment (14). These system-wide health benefits, which are not captured in ophthalmic models, further strengthen the case for oxygen blending as a foundational neonatal safety intervention. Unblended 100% oxygen may save some infants in the short term, but it also increases mortality and causes preventable injury to the retina, lungs, and brain; in contrast, blended, titrated oxygen saves more infants with less blindness and less long-term disability, preventing a generation of oxygen-injured survivors.

The burden of lost productivity due to ROP is clear. Marques et al. estimated that worldwide, blindness and moderate/severe visual impairment led to an overall employment reduction of 30.2% among people with vision loss (48). As there is a lack of employment opportunities and infrastructure for the blind in Uganda (49), the reduction may be much greater in this context. In Uganda, the annual GDP per capita was $1,002.30 in 2023 (50), and in this study, the cost to avert one DALY is $4.97–$104.98, with a base case of $32.60 if using the oxygen blender that requires medical air and $16.52 if using the oxygen blender that does not. The least favorable scenario ($104.98) is roughly 10% of the annual GDP per capita. In comparison to OME, prevention of malaria, the leading cause of child mortality in Uganda, via mosquito net distribution was estimated in a 2020 meta-analysis to cost $50 per DALY averted and $2,143 for every death prevented (51). We estimated the cost to prevent one case of SVI with OME to be between $25.55–$543.99, with a base case of $168.92 if using the oxygen blender that requires medical air and $85.33 if using the oxygen blender that does not.

Other highly cost-effective public health interventions include vitamin A supplementation, shown to have a cost-utility of $11–$12 per DALY averted in Nepal (52), and oral rehydration therapy, shown to have a cost-utility of $214 per DALY averted in Myanmar (53). We acknowledge that the $4.97 per DALY averted in this study is a very low figure, but we note that this is the most favorable scenario, meaning that all clinical and cost parameters are at their maximally advantageous values; such a scenario is unlikely. Extremely cost-effective interventions in Uganda have been studied previously; a 2011 report found that a maximally cost-effective strategy of measles vaccination in Uganda would cost $1.50 per DALY averted (54). In this study, the base case scenarios are more likely to be reflect the true cost-effectiveness, but we aimed to provide a range of plausible values.

**Figure 5:**
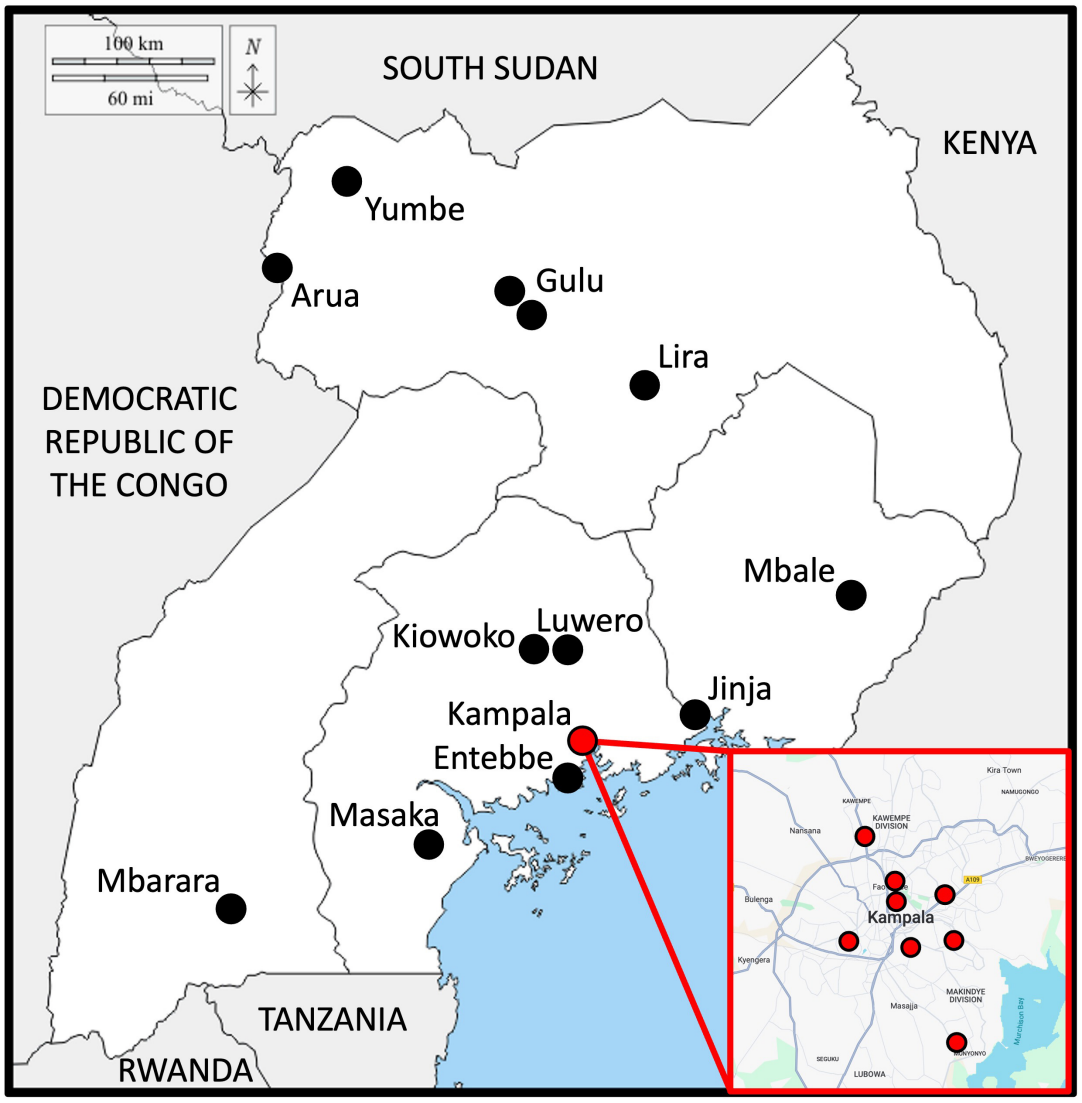
Neonatal intensive care units in Uganda. 20 of the neonatal intensive care units in Uganda. There are multiple neonatal units in Kampala, marked in red. (Original maps courtesy of https://d-maps.com/carte.php?num_car=4022&lang=en and Google Maps). Lower-acuity health facilities, which also administer 100% oxygen to premature infants, are not included here.

Many new healthcare initiatives in SSA are funded wholly or in part through philanthropy. However, for non-communicable conditions such as visual impairment, this is often not the case. Though these conditions represented 67% of worldwide DALYs in 2023, just 2.32% of all global health financing went toward non-communicable diseases in that year, whereas 30.79% went toward HIV/AIDS, malaria, and TB combined which accounted for 7% of all DALYs (55–57). Flows of development assistance are also slowing in the post-pandemic era (58). Thus, public funding and public-private partnerships would likely be critical to the success of ROP prevention, screening, and treatment in the 21 neonatal intensive care units and other health facilities throughout Uganda that are now treating preterm newborns with oxygen (Figure 5). Collaboration between for-profit and non-profit hospitals in the fields of training and equipment sharing should also be explored (59). Lost productivity due to visual impairment within Africa is well-studied, and in any given country experiencing the third wave of ROP, it is within that country's interest—both financial and humanitarian—to comprehensively address it (60, 61).

Limitations of this study include potential discrepancies between the estimates of values used in this analysis and the actual values related to equipment purchased, as well as estimates of the number of preterm infants potentially at risk due to exposure to unblended oxygen. Additionally, some estimates were derived from limited datasets; the turnover rate of oxygen blenders, for example, has only been studied at two hospitals in Uganda (18). In that study, median and IQR were reported. We implemented an analysis of most and least favorable scenarios to capture a broader range of estimated cost, but the possibility remains that the true cost-effectiveness lies outside the range that we determined. Additionally, there are factors that affect cost-effectiveness that we did not include in order to simplify this study. These include costs of training, installation/maintenance costs, and some required disposables.

In our previous study in Kampala, installation and maintenance costs were absorbed by the general hospital budget (24), but learning how to use the OME and appropriately titrate oxygen delivery does require training. Upfront training and ensuring that a NICU has appropriate nursing ratios to enable delivery of blended oxygen therapy will likely represent some of the most difficult aspects of OME implementation. In our intervention in Kampala, training of the staff at St. Francis Nsambya Hospital was completed by the Head of Pediatrics (VN) with support from the Stop Infant Blindness in Africa initiative, as acknowledged in that manuscript (24), and TinyEyes (tinyeyes.org). For this analysis, figures for training costs were not specifically available and were not included. The availability of medical air at St. Francis Nsambya Hospital in Kampala enabled use of a medical air/oxygen blender (Neotech). However, medical air is not available in most neonatal care units in Uganda, limiting the current country-wide generalizability of medical air/oxygen blender use. Therefore, we have included a second base case analyzing the Vayu blender as an alternative since this newer, low cost device uses ambient air rather than medical air to reduce nasal cannula oxygen concentrations (30), broadening the scope of sites where OME could be implemented. The Vayu blender is designed to be used with the Vayu low-flow nasal cannula delivering less than or equal to four liters per minute. While many preterm infants require only low-flow, nasal cannula oxygen, some, including more immature preterm infants, require Continuous Positive Airway Pressure (CPAP), necessitating higher gas flows (>6 L/min). Although a Vayu bCPAP system with an inline blender is available, we did not include it in this analysis as it does not closely align with the use case of the status quo scenario (100% oxygen delivered by nasal cannula) or primary base case scenario (blended oxygen using medical air delivered by nasal cannula). Some neonatal units in SSA do have other devices (e.g., oxygen concentrators, ventilators, CPAP machines) that blend ambient or medical air with oxygen. In the future, there may be less expensive options for delivering blended oxygen via nasal cannula which would substantially increase the cost-effectiveness of intervention with OME.

In addition to the limitations related to OME, we made several assumptions related to treatment based on data from high-income countries than may be difficult to generalize to LMICs. In a 2020 survey of ophthalmologists in SSA, less than half of respondents had access to laser treatment for ROP, but 89% had access to anti-VEGF drugs (15). In our experience, bevacizumab remains the current primary treatment offered by most Ugandan ophthalmologists at this time. Used widely for diabetic retinopathy in SSA, this treatment modality is currently much more accessible than laser. In the literature, treatment success rates with bevacizumab vary between 85% (ROPIC) (26) and 96% (BEAT-ROP) (27) of infants. As noted in Materials and Methods, we used a success rate of 90% in this study, calculated by averaging these two studies. (We note that the 85% figure from the ROPIC study comes from the sensitivity analysis looking at injection-only retreatment, without laser to prevent or treat reactivation.) However, when considering reactivation after anti-VEGF, the short timeframe to 54 weeks' postmenstrual age (BEAT-ROP) overestimates long-term success—the 96% treatment success rate has likely fallen over time. The ROPIC study had longer follow-up, likely adequate to identify most cases of reactivation, and it demonstrated a more realistic success rate of 85%. The study showed that many infants will need retreatment and could be missed without close follow-up (26). However, we do not know the natural history of reactivated ROP that goes untreated. In this study we assumed that 50% of infants with treatment-warranted ROP who reactivate after primary treatment and do not follow up for reactivation will progress to SVI. The 10% rate of reactivation after bevacizumab, which we use for this analysis, does not describe rates of reactivation after treatment with ranibizumab, aflibercept, or biosimilars.

However, an 85% success rate may actually represent a significant underestimation: both reactivation and persistent avascular retina (PAR), two complications of treatment, counted toward the complementary 15% failure rate. In Uganda, there is a lack of long-term follow-up data for anti-VEGF treatment in infants, and it may be possible that reactivation and PAR occur at lower rates because the larger, healthier infants affected by the third wave of ROP may vascularize more completely following treatment when compared to their low-birth weight and low-gestational age counterparts in the ROPIC study (21, 26). Also, not all reactivations will lead to adverse outcomes—some cases may regress on their own, and this rate is unknown (we assume 50%). In particular, the reactivation rate of aggressive ROP (AROP), in the setting of unblended oxygen in larger, healthier preterm infants who may vascularize more completely, is unknown (3, 21). Evaluation for reactivation and laser treatment completion will remain important in SSA as it will in high-income countries (62).

Additionally, we estimated that 62.1% of all the cases of severe ROP will be severely visually impaired or blind with no treatment, based on the natural history of untreated eyes in the CRYO-ROP study (25, 31). However, the true rate is likely lower because Type 1 disease—the stage that now represents treatment criteria—includes some pre-threshold disease and threshold disease, only the latter of which was the treatment criteria used in CRYO-ROP. In short, earlier stages of ROP now meet treatment criteria, so a smaller proportion of infants with treatment-warranted ROP who remain untreated will progress to SVI. Because treatment has been successful since CRYO-ROP, subsequent data on the natural history of untreated ROP is limited. Overall, the use of this figure in our analysis over-estimates the impact of OME.

We assume a 70% deployment rate of OME in our analysis of a hypothetical national program—that is, 70% of the 35,865 surviving at-risk premature infants in a one-year birth cohort would receive blended oxygen in a neonatal unit. The annual number of at-risk infants who are never admitted to a hospital in Uganda is unknown, but is likely an appreciable portion of this annual cohort. This is intended to reflect possible barriers to distributing OME to every neonatal unit in the country, as well as to account for high-risk preterm infants who receive 100% oxygen in a non-neonatal unit. We also used the 54% screening rate observed at the major, large public hospital in Kampala. This number is likely different at other hospitals and may be zero at some and higher at others. Lastly, we assumed that in hospitals that have OME, the OME will be utilized nearly 100% of the time. This assumption definitionally accepts some blindness, as near-100% OME utilization means that at least some premature infants will receive unblended oxygen. However, if implemented well, the highest-risk infants in a given NICU would be the first to receive blended oxygen, and lower-risk infants who receive unblended oxygen during days with insufficient inventory would require oxygen (and be exposed to unblended oxygen) for a short amount of time, compared to higher-risk infants.

Importantly, in SSA, the WHO has also estimated that over half of blind children will die within one to two years of becoming blind (33). If this figure had been included in this analysis, it would increase the number of DALYs averted for each infant in which ROP is prevented. However, this estimation is neither specific to blindness from ROP or to Uganda, and many of the deaths are likely due to other sequelae of prematurity. Therefore, for the purpose of this analysis we assumed that each infant would live to 58 years old—the average lifespan in Uganda. Additionally, to further simplify our study, we assumed that no infants with SVI would progress to blindness, which has a more severe disability weighting. Incorporating progression to blindness would therefore increase our estimated cost-effectiveness. The impact of SVI and blindness extends beyond vision to the ability to learn and get an education, as well as the opportunity cost of lost work for caregivers, none of which are included in this analysis. Therefore, we assume that the downstream impacts of vision loss on a child and the family significantly outweigh the costs of maintenance and training for the OME. Lastly, as mentioned previously, we only account for the cost-effectiveness of OME as it pertains to ROP in this study. However, there are other complications associated with administration of 100% oxygen and subsequent hyperoxia, including bronchopulmonary dysplasia and intraventricular hemorrhage (14). Including these complications, which may be prevented with OME, into our analysis would likely result in an increase in the calculated cost-effectiveness. The disability weight for SVI due to ROP is 0.184. To briefly compare this to other pediatric conditions: severe congenital hearing loss, moderate intellectual disability, and “moderate COPD and other chronic respiratory problems” (included here to represent bronchopulmonary dysplasia) have disability weights of 0.158, 0.100, and 0.225, respectively, with a DW of 0 indicating perfect health and 1 indicating death (32).

Finally, we assume 69.8% survival among preterm infants, as reported in a 2020 Ugandan study (20), the only published rate in a Ugandan setting. However, we note that this study included all infants < 37 weeks' gestation, so it likely underestimates mortality among infants < 34 weeks' gestation. A 14.9% rate of progression to SVI due to ROP in infants treated with unblended oxygen was observed at St. Francis Nsambya Hospital in Kampala. The survival rate there is >95%, but this is likely higher than the average rate across Uganda, as the mortality rate is dependent upon the resources available at a specific site, among other factors. A 2025 meta-analysis estimated low birth weight mortality across SSA to be 33.1% (63), though many of the studies included were several years old and likely do not reflect recent advances in care. Improving neonatal care across the country will continue to lead to improved survival of progressively more immature preterms and subsequently increased rates of ROP when unblended oxygen is used.

In conclusion, it is well-understood that prevention, when possible, is generally more efficient than treatment as a strategy for addressing high-incidence and high-cost diseases. Until now, the cost-effectiveness of preventive strategies for ROP had not been explored in SSA. We chose to examine Uganda due to our experience in the country, but the conclusions we reached regarding cost-effectiveness apply to a wider context, although exact costs will vary by country. The implementation of OME is a very cost-effective means to reduce the incidence of severe ROP in Uganda and would consequently reduce the burden of SVI on infants throughout SSA and in LMICs globally.

## Data Availability

The original contributions presented in the study are included in the article/supplementary material, further inquiries can be directed to the corresponding author.
